# Prevalence and association of musculoskeletal disorders with various risk factors among older Indian adults: Insights from a nationally representative survey

**DOI:** 10.1371/journal.pone.0299415

**Published:** 2024-10-23

**Authors:** Jaya Tiwari, Pritam Halder, Divya Sharma, Uttam Chand Saini, Vineeth Rajagopal, Tanvi Kiran

**Affiliations:** 1 Department of Community Medicine and School of Public Health, PGIMER, Chandigarh, India; 2 Department of Orthopaedics, PGIMER, Chandigarh, India; Vector Control Research Centre, INDIA

## Abstract

**Background:**

Globally, Musculoskeletal disorders (MSDs) are the biggest contributor (17%) to years lived with disability. For offering diagnosis and appropriate health interventions options along with follow-up care, it is pertinent to have a thorough awareness of its associated risk factors.

**Objectives:**

The study aims to assess the prevalence and association between MSDs and risk factors among the Indian older adults above 45 years.

**Methodology:**

Prevalence of MSDs among 28,436 participants was estimated using nationally representative survey on older Indian adults. Spatial distribution maps were created to display the magnitude of MSD prevalence at Indian sub-national level. Association between presence of MSDs and associated risk factors (such as occupation, employment duration, physical activity, BMI, diabetes, hypertension, tobacco usage, and alcohol consumption) was explored through Multivariate logistic regression. P-value <0.05 was considered as statistically significant.

**Results:**

MSD was prevalent in over half of participants (53.5 (52.9–54.1)%), with more among females and in those aged >60 years (60.4 (59.3–61.4)%). Overall, joint pain (41.9 (41.3–41.4)%) was more common than back pain (32.6 (32.0–33.1)%). Prevalence was highest in Manipur (81.1 (77.9–83.9)%) and lowest in West Bengal (33.1 (30.7–35.5)%). MSD presence was positively associated with certain occupational groups, pre-obesity (BMI 25–29.9 Kg/m^2^), currently hypertensive, vigorous physical activity among overall population. Tobacco usage was positively associated, while alcohol consumption was linked to a lower occurrence of MSDs for 45–60 years age group.

**Conclusions:**

Customised policy interventions can be developed for specific age category of older Indian adults and musculoskeletal health can be improved by addressing modifiable risk factors such as physical inactivity, tobacco usage, avoiding workplace risks in occupations requiring manual labour as revealed by this study.

## Introduction

Various illnesses that affect the locomotor system, including the muscles, bones, joints, and tendons, are referred to as musculoskeletal disorders (MSDs). Common forms of MSDs include rheumatoid arthritis (RA), osteoarthritis (OA), low back pain (LBP), neck pain (NP), and gout [1]. Joint pain, stiffness, and decreased mobility are the characteristics of these illnesses, which can result in physical dysfunction, depressive symptoms, and other chronic health issues like cardiovascular diseases [2].

As per Global Burden of Disease data, MSDs accounted for 17% of all years lived with disability (YLDs) worldwide in 2019, placing them as the most significant contributor to YLDs globally [3]. MSDs frequently result in an elevated risk of all-cause mortality and the emergence of chronic diseases in addition to pain, functional disability, and work incapacity [4]. Even the societal impact of pre-mature retirement due to MSDs is huge with respect to direct health-care costs and indirect costs (absence from work or productivity losses). However, due to their low case fatality rate and irreversibility, MSDs were given less priority than other causes including malignancies and cardiovascular diseases [1]. China, India, and the United States have the most significant incident cases, which increased notably in the 35–64 age group, with the peak shifting from age 35–39 years in 1990 to age 45–49 years [1]. As per the research evidence, MSD prevalence is rising in low-and middle-income (LMIC) nations. Despite this, MSDs were not included in the majority of global NCD initiatives worldwide [5].

In developing nations, the elderly population is expanding quickly and by 2050, according to projections, 79% of people in the globe who are 60 or older will reside in developing countries. Further, older adults are more likely to experience chronic health conditions and impaired health-related quality of life due to physical inactivity, obesity, hypertension, and other non-communicable risk factors [6]. With rising age, there is a tendency towards decrease in body weight or skeletal muscle mass, which may cause a decline in muscle strength, thereby making the older adults susceptible to several MSDs related health issues. The problem of Obesity among Indian older adults also contributes towards MSDs [7]. The prevalence of MSDs among Indian adults’ ranges between 7% to 77% as per the research evidence and as the Indian population ages, it is anticipated that the prevalence of MSDs, which is a major cause of physical disability in older adults, would noticeably increase [5].

A sizable fraction of adults who have one or more MSDs go undiagnosed, either because of inadequate knowledge of the numerous risk factors involved or inadequate possibilities for screening and treatment. Additionally, achieving Sustainable Development Goal-3 (SDG-3), which is "good health and well-being," depends on providing better healthcare services to aging population of the nation. Therefore, encouraging healthy aging is a serious priority, especially in developing nations like India. To diagnose MSD early and offer appropriate health interventions options along with follow-up care, it is necessary to have a thorough awareness of the associated risk factors. Hence, in view of the above backdrop and increasing burden of musculoskeletal disorders in India, the present study using the nationally representative internationally comparable survey data of Longitudinal Aging Study (LASI India), aims to estimate the prevalence and association of MSDs with various risk factors among older adults (aged ≥ 45 years). The study seeks to estimate the magnitude of prevalence at sub-national level in India and spatially map and categorize them into low, medium, high, and very high prevalence regions. The study further envisages to identify the significant common and distinct risk factors attributable to MSD across 45 to 60 years; 60 years & above and overall Indian adult population (aged ≥ 45 years), based on which customised policy interventions can be developed for the specific age category of older Indian adults aimed at addressing the specific risk factors for MSDs.

## Methods

### Study design

The present study follows a cross-sectional study design, the data for which was extracted from the baseline wave-1 of the Longitudinal Ageing Study in India (LASI), that was conducted between 2017 and 2018 [6]. LASI is the largest longitudinal ageing study ever conducted and the first of its kind in India. This national survey is the product of national and international collaboration between the University of Southern California, the International Institute for Population Sciences, and the Harvard T.H. Chan School of Public Health. In the present study, the LASI cross sectional data pertaining to wave-1 was procured from the website of International Institute for Population Sciences after duly filling in the data request form [8].

### Study setting and participants

LASI data included over 72,000 people across all Indian states and union territories who were 45 years of age or older, as well as their spouses (regardless of age). LASI assesses scientific evidence based on demographics, household economic status, chronic health conditions, symptom-based conditions, functional health, mental health (cognition and depression), biomarkers, healthcare utilisation, family and social networks, social welfare programmes, work and employment, retirement, satisfaction, and life expectations for older Indian adults. The sampling strategy of LASI is explained in its wave-1 report in a comprehensive manner. LASI followed a multistage stratified cluster sample design, with three and four separate stages, respectively, for selecting rural and urban areas. The LASI survey was undertaken through computer-assisted personal interview (CAPI) administered by a trained interviewer (face-to-face) during home visits [9].

### Outcome variable

MSD being the outcome variable, is dichotomous (presence or absence). In this study, MSD was considered to be the presence of persistent or troublesome problems of pain or stiffness in joints and/or back pain or problems faced in the past two years. The joint or bone diseases were self-reported and diagnosed as assessed through the LASI question, “HT229- Have you had any of the following persistent or troublesome problems in the past two years?—pains or stiffness in joints; back pain.

### Explanatory variables

Various risk factors for MSD were taken as independent variables. These were categorical variables (occupation, employment duration, vigorous physical activity, BMI) and dichotomous (yes/no) variables (diabetes, hypertension status, tobacco usage, alcohol consumption). Occupation was included as a polychotomous variable having the following categories- legislators and senior officials; professionals; technicians and associate professionals; clerks; service workers and shopkeepers (Employees offer personal and protective services for travel, housekeeping, catering, personal care, and protection from fire and illegal activity, serve as models for artistic creation and display, demonstrate and sell products in retail and wholesale stores, as well as at stalls and markets); skilled agriculture and fishery workers (a certain degree of excellence and necessary proficiency obtained by rigorous technical or professional training or long-term practical work experience; both manual and supervisory work); craft and related trade workers; plant and machine operators; elementary occupations (basic, regular jobs that primarily demand for the use of portable instruments and frequently involve manual labour); and others [10]. Employment duration was categorised as less than five years and more than equal to five years. Vigorous physical activity was categorised as every day; more than once/week; once/week; 1–3 times/ month, and never. BMI was calculated from the given height and weight data (BMI = weight in kg/ height in square meters). BMI (Kg/m^2^) was categorised as <18.5 (undernutrition); 18.5–22.9 (normal); 23–24.9 (overweight); 25–29.9 (pre-obese); and ≥30 (obese) [11].

### Missing value analysis and data pertaining to present study

**Fig 1:**
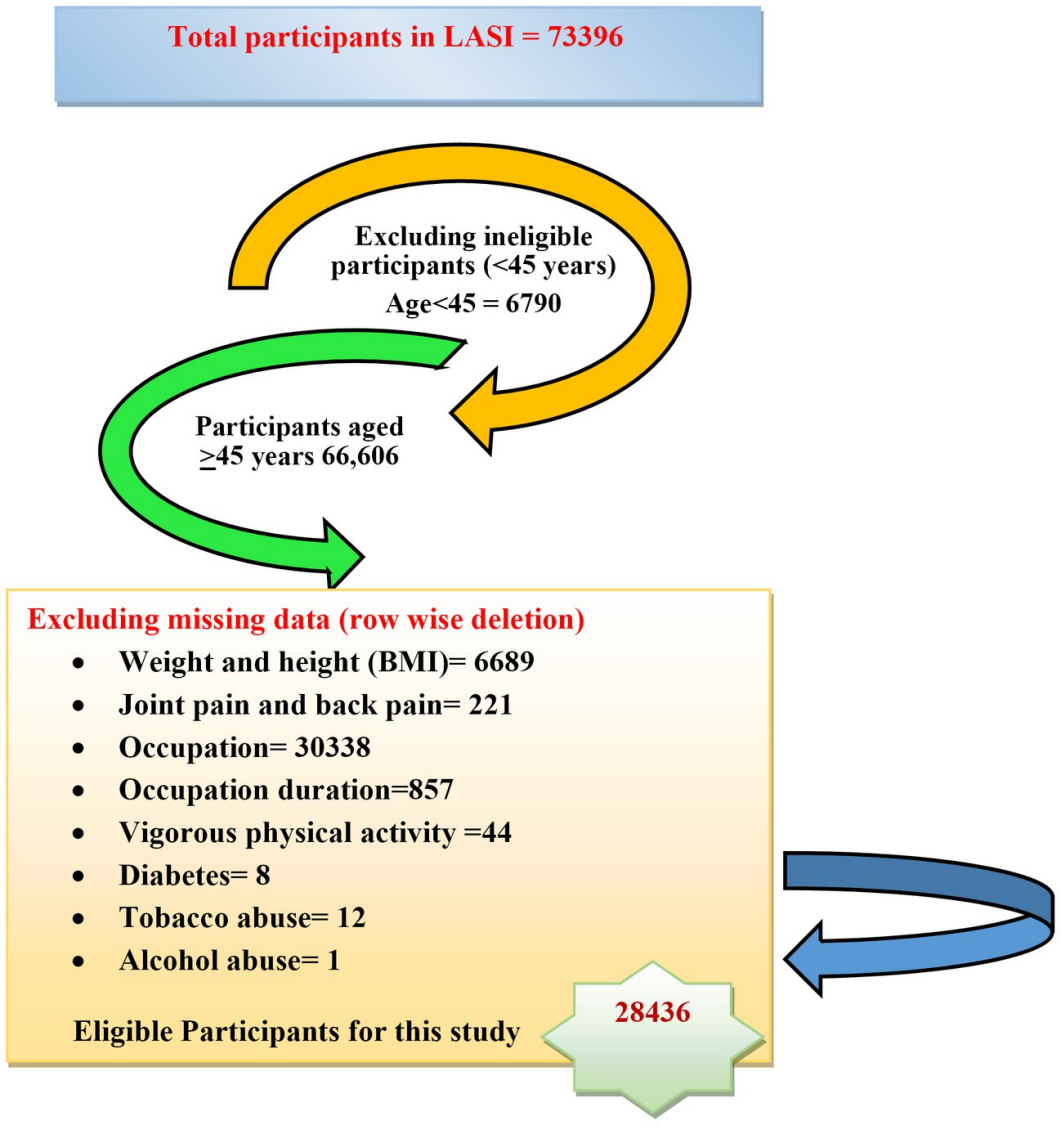
Flowchart showing participants’ selection process in this study.

The LASI wave-1 data includes information of Indian older adults aged 45 years and above or older, including their spouses (regardless of age). The age 45 is selected by LASI keeping in mind its critical significance to examine ageing and social, economic and health transition from the prime adult ages as well to standardize and harmonize the LASI survey for international comparisons. In this backdrop, to perform the current study, we have removed data of participants under the age of 45 years. After adjusting the missing data for all the variables of interest (both outcome and explanatory variables) by row wise deletion, we have included participants aged 45 to 60 (71.7%) and aged > 60 (28.3%) who had documented their MSD status. Details are provided in Fig 1. Thus, the present study based on LASI-first wave data included information from over 28,436 participants. Accordingly using this data, the prevalence of MSD was calculated by presence of joint pain and/or back pain in the last two years.

#### Statistical analysis

**Fig 2:**
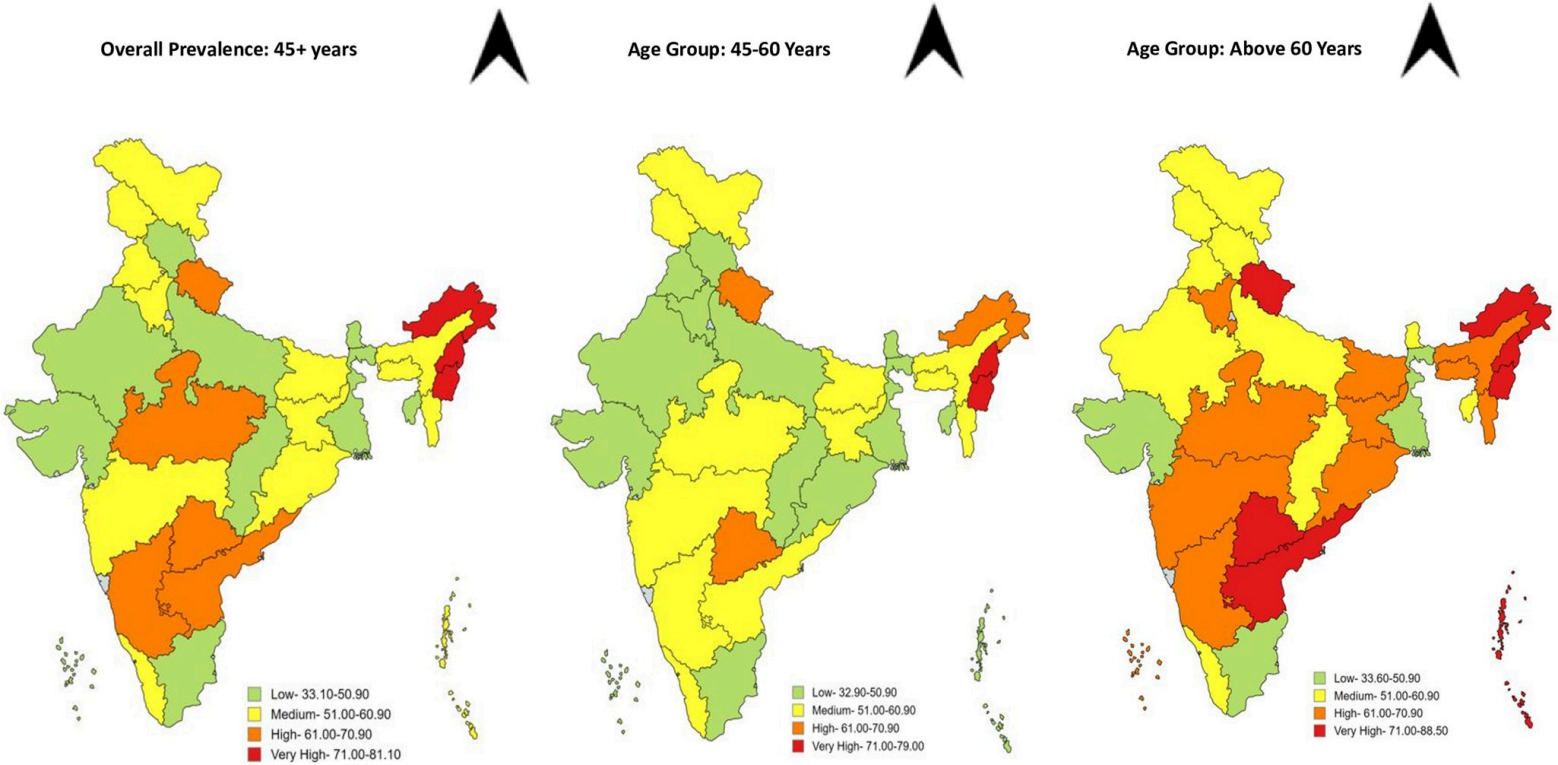
Spatial Mapping and categorization of Indian states/UTs as per MSD prevalence. The maps were created using QGIS version 3.28.9.—https://www.qgis.org/en/site/about/index.html. Shapefiles were used from Survey of India Digital Products- https://www.mapchart.net/india.html. Map imagery sourced from MapChart (https://mapchart.net) under a CC BY license, with permission from Minas Giannekas, original copyright 2024.

Data was analysed in Stata version 17 (Stata Corp. 2017. Stata Statistical Software: Release 17. College Station, TX: Stata Corp LP.). Characteristics of participants were described as mean (standard deviation) for continuous variables; frequencies and percentage for categorical variables. The MSD prevalence was calculated using the eligible participants for the study (n = 28,436). Employing the Quantum Geographic information system (QGIS) and following its equal count approach, all the Indian states/UTs (Union Territories) were classified into low, medium, high, and very high MSD prevalence rates (cut off values) using QGIS version 3.28.9, adding a spatial dimension to the analysis [12, 13]. Different cut off values were generated for each age category shown in Fig 2.

The duration of occupation, vigorous physical activity, BMI, diabetes, hypertension status, tobacco usage, alcohol consumption was taken as explanatory variables. The p value <0.05 was considered statistically significant. Univariate binary logistic regression was conducted with each risk factor. Subsequently, to adjust and account for other risk factors, multivariable logistic regression was also applied separately for older adults aged 45 to 60 years; for above 60 years and for the overall population of older adults (aged 45 and above). The goodness of fit for regression models were reported through overall omnibus test of model coefficients and predictive model classification accuracy rate.

### Ethics

The original LASI study reports compliance with research ethics. Informed written consent was obtained from the participants. Ethical clearance was permitted by the Indian Council of Medical Research (ICMR) and each collaborating bodies [9, 14]. Current study is based on secondary data analysis of Longitudinal Ageing Study in India (LASI) Wave- 1, India [7].

## Results

### Prevalence of MSD and descriptive characteristics

The overall mean (SD) age of the participants was 56.1 (8.6) years. The mean (SD) BMI value (Kg/m^2^) was more in participants aged 45–60 years (22.8 (4.3)) than >60 years (21.4 (4.0)). The overall prevalence of MSD was 53.5% (95% CI: 52.9–54.1)%, and it was observed to be more in participants aged >60 years (60.4% (59.3–61.4)%) than 45–60 years (50.8% (50.1–51.5))%. Prevalence of MSD was significantly (p<0.05) more in females (62.3% (61.4–63.2)%) than males (48.9% (48.1–49.6)%). Both joint pain and back pain were more prevalent among participants aged >60 years than 45–60 years. The overall prevalence of arthritis, rheumatism, osteoporosis, and any bone/joint disease were 6.5% (6.2–6.8)%, 3.7% (3.5–4.0)%, 2.5% (2.3–2.7)% and 11.5% (11.1–11.9)%, respectively and it was relatively more in participants aged >60 years than 45–60 years age group (S1 Table).

### Distribution and mapping of MSD prevalence at sub-national level in India

Prevalence of MSD was highest among participants of Manipur aged ≥45 years (81.1% (77.9–83.9)%). Participants from Nagaland (88.5% (83.4–92.2)%) and Manipur (79.0% (75.0–82.5)%) had highest prevalence of MSD across >60 years and 45–60 years, respectively. Overall, lowest prevalence of MSD was seen in West Bengal (33.1% (30.7–35.5)%). In most of the states, MSD prevalence was much higher in >60 years than 45–60 years age group. But opposite scenario was seen in Jammu and Kashmir. Almost similar MSD prevalence was seen in Gujarat, Mizoram, Tripura, and West Bengal. All the states and union territories were categorised as low, medium, high, very high MSD prevalence and shown as per QGIS mapping (≥45 years/ overall cut off values: low (33.1–50.9%), medium (51.0–60.9%), high (61.0–70.9%) and very high (71.0–81.1%); cut off values for 45–60 years: low (32.9–50.9%), medium (51.0–60.9%), high (61.0–70.9%) and very high (71.0–79.0%); cut off values for >60 years: as low (33.6–50.9%), medium (51.0–60.9%), high (61.0–70.9%), very high (71.0–88.5%)). Among overall population, Chandigarh, Chhattisgarh, Dadra and Nagar Haveli, Daman and Diu, Goa, Gujarat, Himachal Pradesh, Karnataka, Lakshadweep, Puducherry, Rajasthan, Sikkim, Tamil Nadu, Tripura, Uttar Pradesh and West Bengal were categorised as low (L); Andaman and Nicobar, Assam, Bihar, Delhi, Haryana, Jammu and Kashmir, Jharkhand, Kerala, Maharashtra, Meghalaya, Mizoram, Odisha and Punjab were categorised as medium (M); Andhra Pradesh, Madhya Pradesh, Telangana and Uttarakhand were classified as high (H); Arunachal Pradesh, Manipur and Nagaland were categorised as very high (VH) MSD prevalent states/ union territory (Table 1) (Fig 2).

**Table 1:** Distribution of MSD prevalence at sub-national level in India (States/UTs).

State/ UTs	Overall			45–60 years			>60 years		
	Total N = 28436	MSD		Total N = 20396	MSD		Total N = 8040	MSD	
		N = 15213			N = 10360			N = 4853	
		N	%		N	%		N	%
Andaman and Nicobar	306	165	53.9 (M)	251	125	49.8 (L)	55	40	72.7 (VH)
Andhra Pradesh	1132	718	63.4 (H)	796	479	60.2 (M)	336	239	71.1 (VH)
Arunachal Pradesh	548	393	71.7 (VH)	454	315	69.4 (H)	94	78	83.0 (VH)
Assam	884	525	59.4 (M)	692	395	57.1 (M)	192	130	67.7 (H)
Bihar	1494	820	54.9 (M)	932	475	51.0 (L)	562	345	61.4 (H)
Chandigarh	299	140	46.8 (L)	256	114	44.5 (L)	43	26	60.5 (L)
Chhattisgarh	1033	464	44.9 (L)	814	345	42.4 (L)	219	119	54.3 (M)
Dadra and Nagar Haveli	630	288	45.7 (L)	419	184	43.9 (L)	211	104	49.3 (L)
Daman and Diu	344	130	37.8 (L)	254	85	33.5 (L)	90	45	50.0 (L)
Delhi	448	232	51.8 (M)	372	186	50.0 (L)	76	46	60.5 (M)
Goa	307	141	45.9 (L)	235	102	43.4 (L)	72	39	54.2 (M)
Gujarat	1018	398	39.1 (L)	739	282	38.2 (L)	279	116	41.6 (L)
Haryana	578	307	53.1 (M)	452	227	50.2 (L)	126	80	63.5 (H)
Himachal Pradesh	682	342	50.2 (L)	476	231	48.5 (L)	206	111	53.9 (M)
Jammu and Kashmir	314	182	58.0 (M)	229	133	58.1 (M)	85	49	57.7 (M)
Jharkhand	1064	644	60.5 (M)	723	426	58.9 (M)	341	218	63.9 (H)
Karnataka	1110	680	61.3 (L)	740	425	57.4 (M)	370	255	68.9 (H)
Kerala	679	381	56.1 (M)	457	248	54.3 (M)	222	133	59.9 (M)
Lakshadweep	234	102	43.6 (L)	189	74	39.2 (L)	45	28	62.2 (H)
Madhya Pradesh	1257	773	61.5 (H)	918	544	59.3 (M)	339	229	67.6 (H)
Maharashtra	1606	901	56.1 (M)	1096	583	53.2 (M)	510	318	62.4 (H)
Manipur	666	540	81.1 (VH)	462	365	79.0 (VH)	204	175	85.8 (VH)
Meghalaya	401	236	58.9 (M)	309	177	57.3 (M)	92	59	64.1 (H)
Mizoram	556	331	59.5 (M)	414	243	58.7 (M)	142	88	62.0 (H)
Nagaland	604	482	79.8 (VH)	395	297	75.2 (VH)	209	185	88.5 (VH)
Odisha	1226	680	55.5 (M)	872	444	50.9 (L)	354	236	66.7 (H)
Puducherry	494	220	44.5 (L)	342	132	38.6 (L)	152	88	57.9 (M)
Punjab	570	300	52.6 (M)	416	210	50.5 (L)	154	90	58.4 (M)
Rajasthan	940	445	47.3 (L)	667	295	44.2 (L)	273	150	55.0 (M)
Sikkim	201	88	43.8 (L)	159	64	40.3 (L)	42	24	57.1 (M)
Tamil Nadu	1520	540	35.5 (L)	1058	353	33.4 (L)	462	187	40.5 (L)
Telangana	1096	715	65.2 (H)	784	484	61.7 (H)	312	231	74.0 (VH)
Tripura	500	250	50.0 (L)	382	189	49.5 (L)	118	61	51.7 (M)
Uttar Pradesh	1753	845	48.2 (L)	1194	544	45.6 (L)	559	301	53.9 (M)
Uttarakhand	514	343	66.7 (H)	365	229	62.7 (H)	149	114	76.5 (VH)
West Bengal	1428	472	33.1 (L)	1083	356	32.9 (L)	345	116	33.6 (L)
Overall	**28436**	**15213**	**53.50 (M)**	**20396**	**10360**	**50.8 (L)**	**8040**	**4853**	**60.4 (M)**

### Association of related risk factors with musculoskeletal disorder among Indian older adults

**Fig 3:**
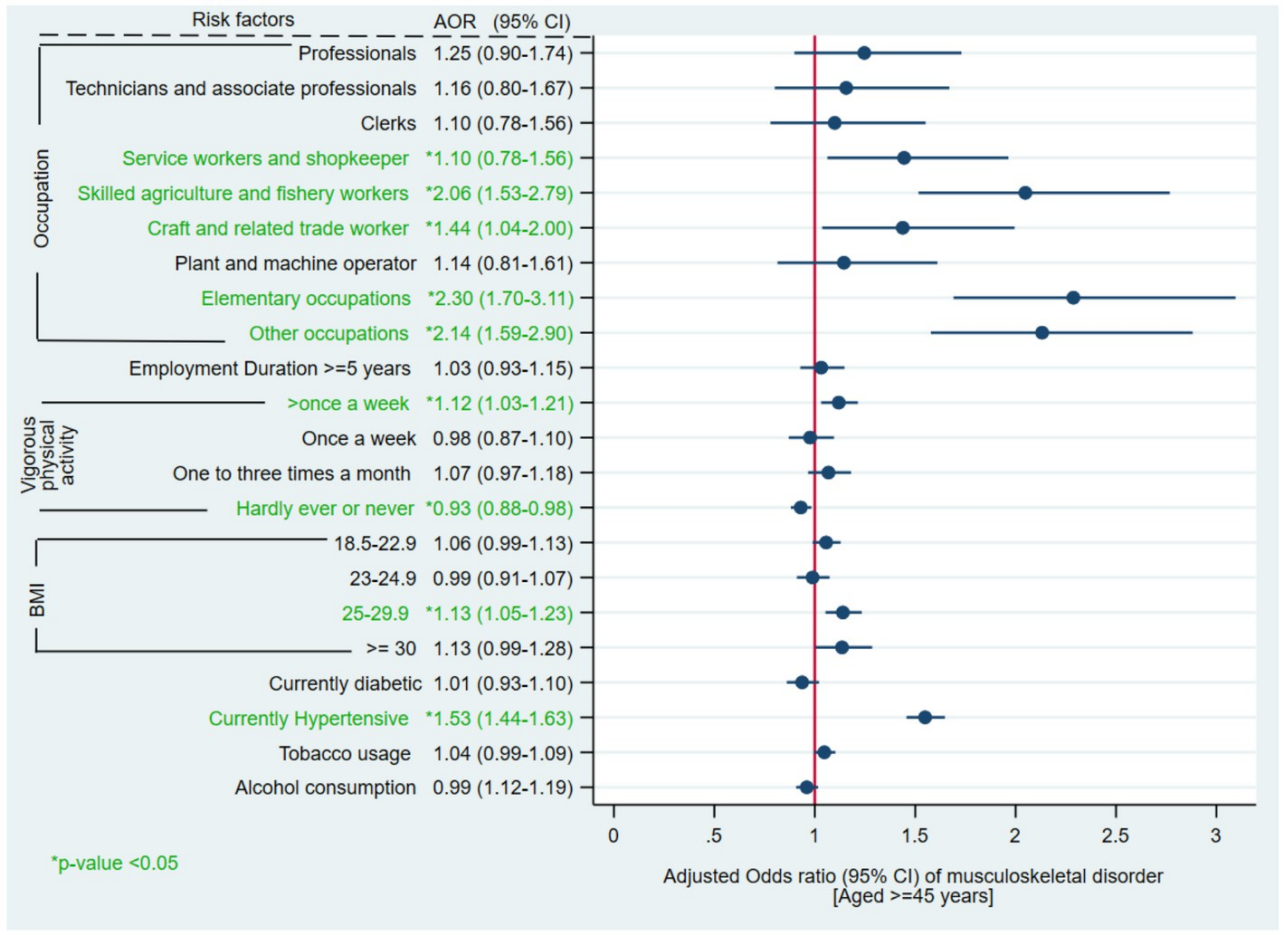
Multivariable logistic regression of musculoskeletal disorders and various risk factors among overall population (≥45 years).

Skilled agriculture and fishery work was the most common occupation overall (36.3%) and in the age subgroups also. Participants with elementary occupation had highest MSD prevalence among overall (57.8%), among 45–60 years (54.4%) and >60 years (66.2%) age group. Lowest MSD prevalence was seen in legislators and senior officials among overall (38.5%), among 45–60 years (38.7%) and >60 years (37.8%) age group. Majority of the participants were employed at least for 5 years. Prevalence of MSD was almost similar irrespective of employment duration. Around 38.5% participants performed vigorous physical activity on everyday basis. While 39.6% participants never performed any vigorous physical activity. Prevalence of MSD was highest among participants who performed vigorous physical activity more than once per week among overall (56.8%) and in other subgroups also. Majority of the participants had BMI 18.5–22.9 Kg/m^2^ among overall (40.8%) and in other age-classes Prevalence of MSD was ranging from 51.6% to 54.9% across different BMI categories. Around 9.3% participants were currently diabetic, relatively more in age group >60 years (11.1%) than 45–60 years (8.6%). Prevalence of MSD was almost similar irrespective of diabetes status across age-classes. Almost 21.1% participants were currently hypertensive, relatively greater in age group >60 years (25.8%) than in 45–60 years (19.2%). MSD prevalence was more in currently hypertensive participants than normotensive overall (61.1%), in age group 45–60 years (59.0%) and >60 years (65.0%). Tobacco usage was 46.5%, more in age group >60 years (51.3%) than 45–60 years (44.6%). Prevalence of MSD was almost similar irrespective of tobacco usage. Around 26.5% participants had history of alcohol consumption, more in age group >60 years (61.8%) than 45–60 years (49.7%). Prevalence of MSD was almost similar irrespective of alcohol consumption (Table 2). Taking the overall view of the older adults (≥45 years), in comparison to legislators and senior officials, the adjusted odds of having MSD were 2.30, 2.14, 2.06; 1.10 and 1.44 times significantly higher for elementary occupations; other occupations; skilled agriculture and fishery workers; service workers and shopkeepers; and craft and related trade workers respectively overall. The odds of occurrence of MSD were significantly 12% higher and 7% lower in participants who performed vigorous physical exercise more than once a week (aOR 1.12, 95% CI 1.03–1.21) and never exercised (aOR 0.93, 95% CI 0.88–0.98), respectively as compared to the older adults who indulged in vigorous physical exercise on everyday basis. Keeping the participants with BMI <18.5 as the reference category, the likelihood of MSD prevalence was 13% significantly higher (p< 0.05) in participants with BMI 25–29.9 (aOR 1.13, 95% CI 1.05–1.23). The odds of occurrence of MSD were 53% significantly higher in currently hypertensive (aOR 1.53, 95% CI 1.44–1.63) than normotensive participants (Fig 3, S2 Table).

**Table 2:** Bivariate association of various related risk factors with musculoskeletal disorder among Indian older adults (≥45 years).

Variable	Total (N = 28436) N (%)	MSD present (N = 15213) N (%)	Chi-square and p-value	Age 45–60 years (N = 20396) N (%)	MSD present (N = 10360) N (%)	Chi-square and p-value	Age >60 years (N = 8040) N (%)	MSD present (N = 4853) N (%)	Chi-square and p-value
**Occupation**									
Legislators and senior officials	187 (0.7)	72 (38.5)	<0.001	150 (0.7)	58 (38.7)	<0.001	37 (0.5)	14 (37.8)	<0.001
Professionals	809 (2.8)	354 (43.8)		691 (3.4)	290 (42.0)		118 (1.5)	64 (54.2)	
Technicians and associate professionals	341 (1.2)	141 (41.4)		298 (1.5)	122 (40.9)		43 (0.5)	19 (44.2)	
Clerks	525 (1.9)	213 (40.6)		449 (2.2)	174 (38.8)		76 (1.0)	39 (51.3)	
Service workers and shopkeeper	2,509 (8.8)	1,175 (46.8)		1,838 (9.0)	824 (44.8)		671 (8.4)	351 (52.3)	
Skilled agriculture and fishery workers	10,311 (36.3)	5,632 (54.6)		6,830 (33.5)	3560 (52.1)		3,481 (43.3)	2072 (59.5)	
Craft and related trade worker	827 (2.9)	383 (46.3)		612 (3.0)	273 (44.6)		215 (2.7)	110 (51.2)	
Plant and machine operator	571 (2.0)	233 (40.8)		482 (2.4)	197 (40.9)		89 (1.1)	36 (40.5)	
Elementary occupations	5,468 (19.2)	3,160 (57.8)		3,896 (19.1)	2119 (54.4)		1,572 (19.6)	1041 (66.2)	
Others	6,888 (24.2)	3,850 (55.9)		5,150 (25.3)	2743 (53.3)		1,738 (21.6)	1107 (63.7)	
**Employment Duration (years) documented**									
<5	1486 (5.2)	778 (52.4)	0.364	1038 (5.1)	511 (49.2)	0.301	448 (5.6)	267 (59.6)	0.734
≥5	26950 (94.8)	14435 (53.6)		19358 (94.9)	9849 (50.9)		7592 (94.4)	4586 (60.4)	
**Vigorous physical activity**									
Everyday	10,945 (38.5)	5912 (54.0)	<0.001	8140 (39.9)	4209 (51.7)	<0.001	2805 (34.9)	1,703 (60.7)	0.246
More than once / week	3,044 (10.7)	1730 (56.8)		2208 (10.8)	1,199 (54.3)		836 (10.4)	531 (63.5)	
Once / week	1,333 (4.7)	708 (53.1)		964 (4.7)	486 (50.4)		369 (4.6)	222 (60.2)	
1–3 times /month	1,849 (6.5)	1029 (55.6)		1290 (6.3)	689 (53.4)		559 (7.0)	340 (60.8)	
Never	11,265 (39.6)	5834 (51.8)		7794 (38.2)	3,777 (48.5)		3471 (43.2)	2,057 (59.3)	
**BMI**									
<18.5	5,329 (18.7)	2,810 (52.7)	0.015	3268 (16.0)	1620 (49.6)	0.068	2061 (25.6)	1190 (57.7)	0.001
18.5–22.9	11601 (40.8)	6,241 (53.8)		8163 (40.0)	4158 (50.9)		3438 (42.8)	2083 (60.6)	
23–24.9	4378 (15.4)	2,260 (51.6)		3345 (16.4)	1652 (49.4)		1033 (12.9)	608 (58.9)	
25–29.9	5730 (20.2)	3,135 (54.7)		4464 (21.9)	2326 (52.1)		1266 (15.8)	809 (63.9)	
≥30	1398 (4.9)	767 (54.9)		1156 (5.7)	604 (52.3)		242 (3.0)	163 (67.4)	
**Currently Diabetic**									
No	25781 (90.7)	13785 (53.5)	0.756	18636 (91.4)	9450 (50.7)	0.424	7145 (88.9)	4335 (60.7)	0.107
Yes	2655 (9.3)	1428 (53.8)		1760 (8.6)	910 (51.7)		895 (11.1)	518 (57.9)	
**Currently Hypertensive**									
No	22459 (78.9)	11561 (51.5)	<0.001	16491 (80.9)	8055 (48.8)	<0.001	5968 (74.2)	3506 (58.8)	<0.001
Yes	5977 (21.1)	3652 (61.1)		3905 (19.2)	2305 (59.0)		2072 (25.8)	1347 (65.0)	
**Tobacco usage**									
No	15225 (53.5)	8080 (53.1)	0.120	11306 (55.4)	5695 (50.4)	0.178	3919 (48.7)	2385 (60.9)	0.375
Yes	13211 (46.5)	7133 (53.9)		9090 (44.6)	4665 (51.3)		4121 (51.3)	2468 (59.9)	
**Alcohol consumption**									
No	20895 (73.5)	11199 (53.6)	0.583	15022 (73.7)	7686 (51.2)	0.077	5873 (73.1)	3513 (59.8)	0.100
Yes	7541 (26.5)	4014 (53.5)		5374 (26.3)	2674 (49.7)		2167 (26.9)	1340 (61.8)	

**Fig 4:**
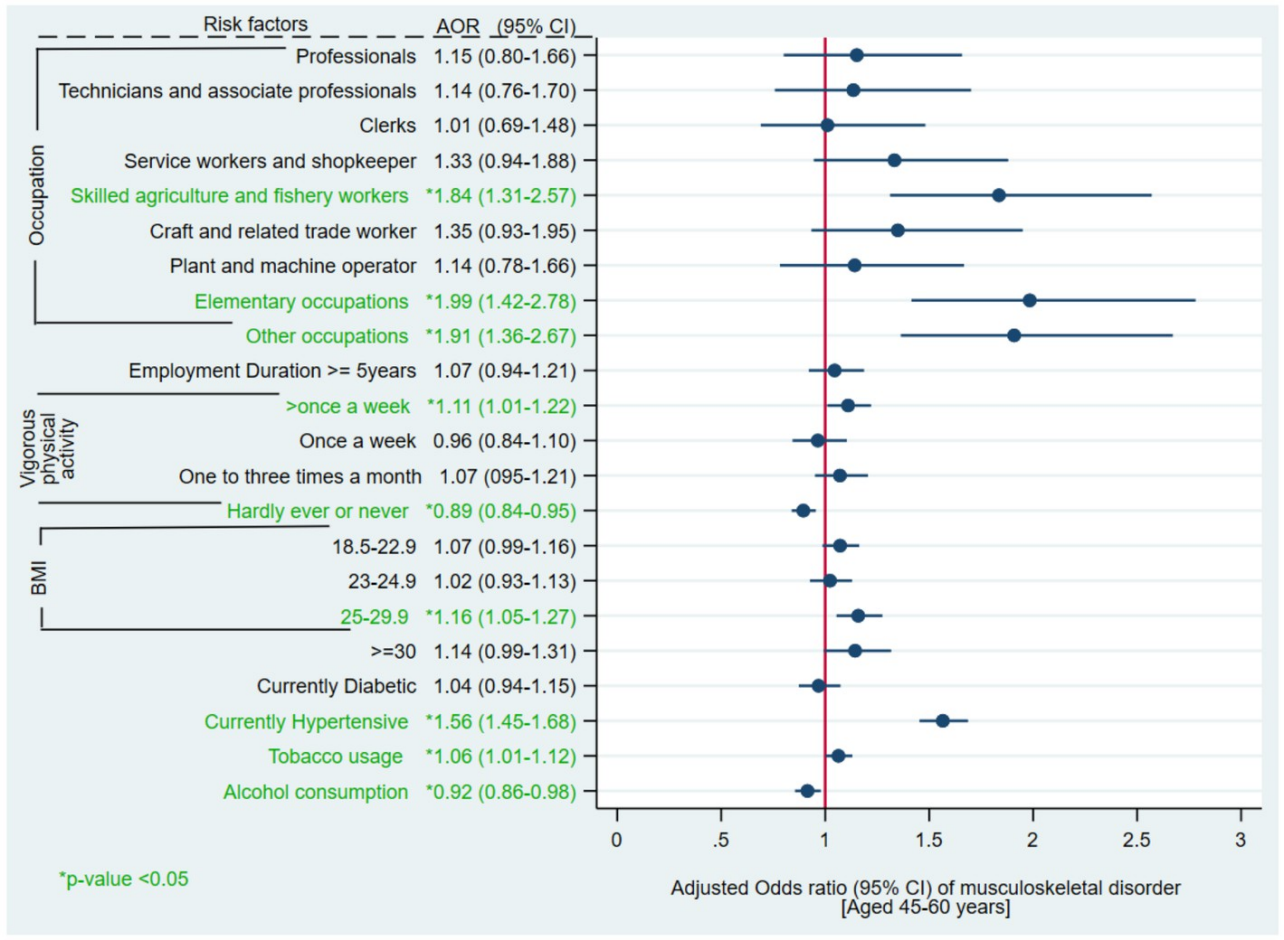
Multivariable logistic regression of musculoskeletal disorders and various risk factors among Indian population aged 45–60 years.

Among participants aged 45–60 years, the adjusted odds of having MSD were 1.84, 1.99 and 1.91 times significantly higher in skilled agriculture and fishery workers (aOR 1.84, 95% CI 1.31–2.57); elementary occupations (aOR 1.99 95% CI 1.42–2.78) and other occupations (aOR 1.91, 95% CI 1.36–2.67), respectively when compared with legislators and senior officials. With respect to participants performing vigorous physical exercise every day, the odds of prevalence of MSD were 11% significantly higher and 11% lower in participants performing vigorous physical exercise more than once/ week and never exercised, respectively. With respect to participants with BMI <18.5; the likelihood of MSD prevalence was 16% significantly higher in participants with BMI 25–29.9 (aOR 1.16, 95% CI 1.05–1.27). The odds of occurrence of MSD were 56% significantly higher in currently hypertensive (aOR 1.56, 95% CI 1.45–1.68) than normotensive participants. The likelihood of MSD was 6% higher in participants with history of tobacco consumption (aOR 1.06 95% CI 1.01–1.12) than non-consumers with statistically significant difference. The participants with history of alcohol consumption (aOR 0.92, 95% CI 0.86–0.98) had significant 8% lower prevalence of MSD than non-consumers (Fig 4, S3 Table).

**Fig 5:**
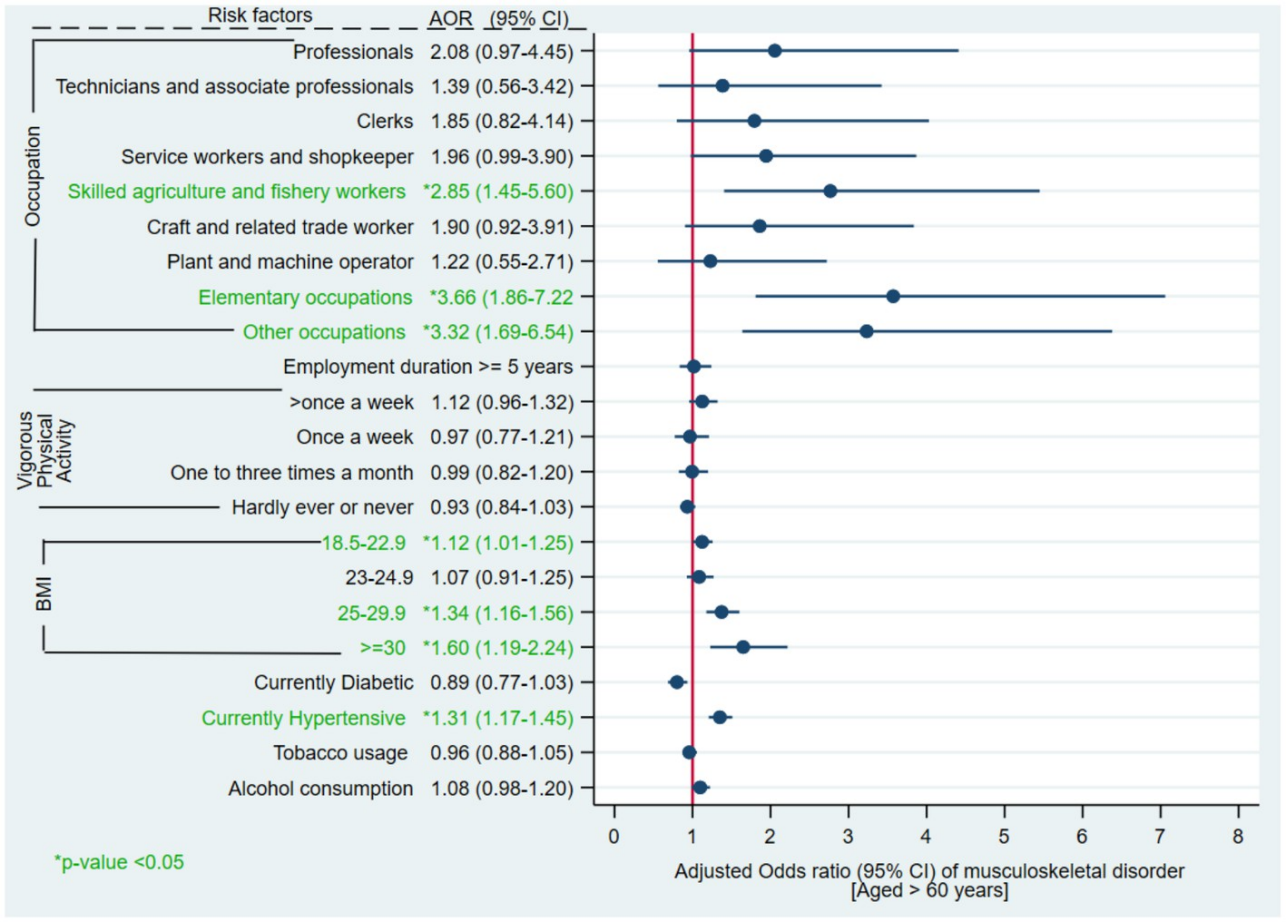
Multivariable logistic regression of musculoskeletal disorders and various risk factors among Indian population aged >60 years.

Among participants aged >60 years, the adjusted odds of having MSD were 3.66, 3.32 and 2.85 times significantly higher in elementary occupations (aOR 3.66, 95%CI 1.86–7.22); other occupations (aOR 3.32, 95%CI 1.69–6.54); and skilled agriculture and fishery workers (aOR 2.85, 95% CI 1.45–5.60), respectively as compared to legislators and senior officials. With increase in the BMI, the odds of MSD occurrence also increase as observed from the results. When compared to BMI <18.5, the likelihood of MSD was 12%, 34% and 60% significantly higher in participants with BMI 18.5–22.9 (aOR 1.12 95%CI 1.01–1.25), 25–29.9 (aOR 1.34, 95%CI 1.16–1.56) and ≥30 (aOR 1.60, 95%CI 1.19–2.24), respectively. The likelihood of MSD occurrence was 31% significantly higher in currently hypertensive (aOR 1.31, 95%CI 1.17–1.45) than normotensive participants (Fig 5, S4 Table).

With regard to goodness of fit statistics for all the three logistic regression models (S2–S4 Tables), the overall omnibus test of model coefficients has come out to be statistically significant. Further, the predictive model classification accuracy has also come out to be more than the cut off value (50%), indicating all three models have reasonable predictive accuracy and good fit [15].

**Fig 6:**
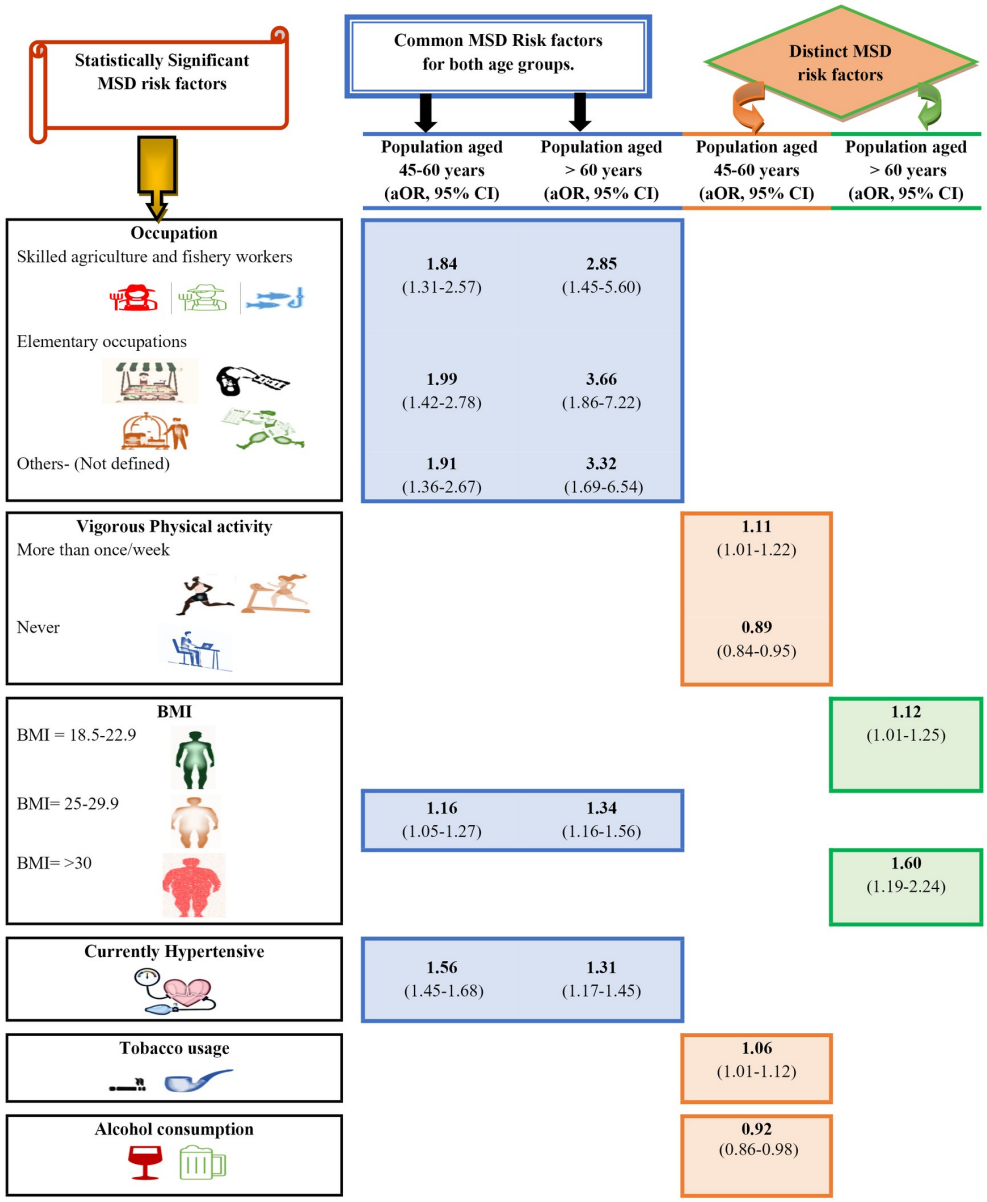
Common and distinct risk factors for MSD for specific age groups of older Indian adult population. *Note- The images in this figure are adapted from Microsoft office 365 power point presentation software and from https://www.istockphoto.com/.

Common and distinctive significantly associated risk factor with MSD occurrence among 45–60 years and >60 years population, common significantly associated risk factor with MSD were occupation (skilled agriculture and fishery workers; elementary occupation and others), BMI (25–29.9), and currently hypertensive. While, among overall and 45–60 years population, common significantly associated risk factors with MSD were elementary occupations and vigorous physical activity (more than once/ week; never). Unique risk factor significantly associated with MSD among overall participants was occupation (service workers and shop keepers (aOr = 1.45 95% CI 1.07–1.97); craft and related trade workers (aOr = 1.44 95% CI 1.04–2.00)). Significantly associated unique risk factors among population aged 45–60 years were tobacco usage (aOr = 1.06 95% CI 1.01–1.12) and alcohol consumption (aOr = 0.92 95% CI 0.86–0.98). Significantly associated risk factor unique among population aged >60 years was BMI (18.5–22.9, ≥30) (aOr = 1.12 95% CI 1.01–1.25) (Fig 6, S5 Table).

## Discussion

A total of 28,436 participants were included in the current study. The study estimated the prevalence of MSDs and found that half of the participants aged 45–60 years had MSD in the last two years. Deshmukh et al. reported a higher prevalence in adults aged 30–60 years in rural Maharashtra [16]. Additionally, the study found that more than half of the participants aged over 60 years were affected by MSDs, a trend similar to the findings was observed by Mendhe et al. in the geriatric population (above 60 years) of rural Andhra Pradesh [17]. In contrast, Sachin et al. reported a relatively lower prevalence of MSDs in the 60–70 age group in Maharashtra and Kirubakaran et al. in rural Tamil Nadu [18, 19]. The current study also reported overall MSD prevalence among almost half of the participants. Lower prevalence rates were documented by Majumdar et al. in adults over 20 years in rural Puducherry, Mathew et al. in rural Kerala for those above 15 years and Bihari et al. in rural Haryana for the 10–70 years age group [20–22]. Additionally, a cross-sectional national survey conducted by Quadir et al. among the adult population in Bangladesh (above 18 years) revealed that 30.4% of participants had MSDs [23]. Meanwhile, a community-based study by Salaffi et al. indicated that the prevalence of MSDs was 26.7% in the adult population in Italy [24].

The prevalence estimated by the current and previous studies unequivocally demonstrates that MSDs are a significant source of disability across all age groups, imposing a substantial burden on individuals and society [5, 14, 15, 19]. Among those under the age of 65, MSDs stand out as the leading cause of disability, and even beyond that age, they remain the second most prominent contributor. Notably, over 50% of reported disabling health issues in adults are attributed to MSDs. These statistics underscore the profound socioeconomic impact of MSD-related conditions. However, it is worth noting that the prevalence of MSDs is more pronounced among elderly adults, primarily because musculoskeletal tissues undergo age-related changes. These changes encompass reduced muscle strength, increased bone fragility, decreased cartilage resilience, diminished ligament elasticity, and altered fat distribution. These age-related alterations compromise the ability of these tissues to perform their typical functions [25].

The prevalence of MSDs was found to be more pronounced in females compared to males in the study. This pattern was consistent with the findings with other studies [18, 19]. The underlying reasons for this gender disparity may be attributed to physiological and anatomical changes, including hormonal variables, body composition, and biomechanics, ultimately leading to functional decline, increased morbidity, and higher mortality rates among older women. These modifications encompass a reduction in overall muscle mass, decreased strength, and coordination in lower limb muscles. Additionally, in older females, post-menopause may raise the risk of developing musculoskeletal conditions like osteoarthritis. This could be associated with oestrogen receptors in articular cartilage, as oestrogen might help preserve chondrocytes before menopause, potentially leading to the development of osteoarthritis [26].

Most of the MSDs are typically marked by pain in the back and joints. According to the present research, approximately one-third of the participants experienced back pain in the preceding two years. A similar outcome was observed in the study conducted by Dar et al. in Kashmir, as well as in the studies by Banerjee et al [27, 28]. Conversely, lower prevalence rates were documented in the research conducted by Mendhe et al. and Kirubakaran et al. [17, 19], whereas Bihari et al. and Dar et al. reported an approximate fifty percent prevalence of back pain [22, 27]. The present study revealed a prevalence of 41.9% for joint pain. Contrasting findings have been reported in other studies, with both higher and lower rates of joint pain documented [18, 19]. This significant variability may be attributed to differences in the study’s population, settings, age groups, gender distribution, occupational factors, physical activity levels, and eligibility criteria, as well as the use of different questionnaires, such as self-developed ones.

MSDs can arise from various underlying risk factors, including occupation, level of physical activity, body mass index (BMI), tobacco or alcohol consumption, and other comorbidities. To determine the association between these factors and the presence of MSDs, the study employed a multivariable logistic regression analysis. The results revealed that individuals in certain occupations, such as service workers, shopkeepers, skilled agriculture and fishery workers, craft and related trade workers, elementary occupations, and others, were more susceptible to MSDs when compared to legislators and senior officials among both 45–60 and above 60 age groups. This increased susceptibility may be attributed to higher stress levels in certain occupations, manual labour, and frequent physical trauma, which can lead to an elevated risk of MSD. Occupations that entail awkward postures, repetitive movements, prolonged standing, increased manual labour, strenuous activities, and extended work hours may also elevate the risk of developing MSD. Numerous other studies have documented similar findings [17].

In the current study, concerning participants engaging in vigorous daily physical activity, MSD was higher among those performing such activities more than once a week in the 45–60 age group. Conversely, among participants who never engaged in vigorous daily physical activity in the same age group, the prevalence of MSD was lower. A study conducted by Banerjee et al. indicated a significant association between MSD and a moderate level of physical activity [28]. Similar findings were observed in the research conducted by Majumdar et al. [20]. This could possibly be attributed to the increased risk of injury associated with higher frequencies of physical activity. Another study by Micheletti et al. demonstrated that engaging in at least five hours of physical activity per week was linked to a reduced risk of MSD [29]. Consequently, varying opinions exist regarding the relationship between regular, intense exercise and musculoskeletal issues. Hasan et al. emphasized the growing prevalence of musculoskeletal disorders and the importance of incorporating non-pharmacological therapeutic strategies, such as exercise [30]. Lastly, Ressinka J et al. underscored the risks of inactivity and the necessity of corrective exercise routines in treating MSDs [31].

Another contributing risk factor for MSDs is the body mass index (BMI) of the person. In individuals with a BMI less than 18.5, it was observed that the occurrence of MSD was notably elevated among those with a BMI in the range of 25 to 29.9 Kg/m^2^, both in the overall population and within specific age groups. Additionally, among individuals aged over 60 years, there was a considerable rise in MSD prevalence with increasing BMI. These findings align with the results reported by Kirubakaran et al. and Majumdar et al. [19, 20]. This trend is likely attributed to unhealthy dietary habits, a sedentary lifestyle, and a lack of physical activity associated with higher BMI, which may contribute to an elevated risk of developing MSD.

It was observed that there was a positive association between hypertension and the prevalence of MSDs in individuals across all age groups. Numerous studies reported similar findings, possibly due to the increased involvement of vascular disorders linked to MSDs [19, 20]. However, a study conducted by Kerkhoff et al. suggested that hypertension did not act as a protective or risk factor for MSDs [32]. Several potential mechanisms could explain the correlation between hypertension and a higher prevalence of pain. According to Chung’s model, individuals with higher blood pressure and greater baroreceptor sensitivity are less likely to experience the wind-up phenomenon, reducing their pain sensitivity. On the contrary, elevated systolic blood pressure may be associated with decreased baroreceptor sensitivity, leading to dysregulated cardiovascular and pain sensitization measures. Altered baroreceptor regulation of circulation not only affects pain perception but also increases the risk of hypertension. People with hypertension and chronic pain tend to exhibit reduced baroreceptor sensitivity and heart rate variability, indicating a role for diminished parasympathetic cardiovascular activity in this connection. The relationship between cardiovascular risk factors and chronic pain may be influenced by common factors such as obesity, physical inactivity, psychosocial variables, proinflammatory cytokines, and the use of nonsteroidal anti-inflammatory drugs [33]. This association may also be modulated by lower parasympathetic cardiovascular activity linked to chronic pain and hypertension in specific individuals [34].

The prevalence of MSDs was found to be higher among individuals with a history of tobacco use compared to those who did not consume tobacco, particularly within the age group of 45–60 years in our study. Smoking has been shown to have adverse effects on the musculoskeletal system, leading to decreased bone mineral density, an increased risk of fractures, bone loss, and a higher likelihood of implant failure, all of which contribute to a greater risk of MSDs. Nicotine, which has a dual impact on bone metabolism, influences bone health through smoking. Higher doses of nicotine tend to slow down bone metabolism, while lower amounts can increase it. Consequently, the detrimental effects of nicotine on bone structure are associated with a heightened risk of lower back pain, increased collagen degradation, and reduced blood and oxygen supply to the bones. Additionally, nicotine may stimulate the production of inflammatory mediators by T-lymphocytes, which can exacerbate musculoskeletal abnormalities and contribute to the development of chronic pain [35].

In the present study, it was observed that individuals with a history of alcohol consumption exhibited a lower prevalence of MSDs than non-consumers, with a statistically significant difference noted within the age group of 45–60 years. Alcohol consumption is known to induce a heightened inflammatory state. Prolonged and heavy drinking can lead to conditions such as rhabdomyolysis, while chronic alcohol addiction may result in proximal myopathy and MSDs. It’s worth noting that a previous study conducted by Micheletti et al. did not find any association between alcohol intake and MSDs [29]. The negative association between alcohol consumption and MSDs observed in the current study might have been coincidental or could be linked to other factors contributing to MSD that were not considered in this research, such as a family history of genetic disorders [36].

### Strengths

The findings from this study can be generalised and applied broadly since LASI serves as a nationally representative survey. Important insights on the prevalence and associational risk factors of musculoskeletal diseases (MSDs) in the Indian context is provided by this research. Notably, this work stands out for its unique approach in estimating and presenting subnational MSD prevalence in India, utilizing LASI secondary data and generating spatial distribution maps through QGIS software. These maps categorize different Indian states into low, medium, high, and very high prevalence categories, shedding light on the extent of the issue, a perspective not explored or presented in prior studies.

The study’s results reveal a significant connection between MSDs and various lifestyle risk factors across younger adults (45–60 years) and elderly (>60 years). Additionally, the research offers a concise overview of the underlying pathophysiological mechanisms associated with these risk factors. Gaining a deeper understanding of these mechanisms could potentially lead to improved clinical outcomes and reduced chances of chronicity for individuals experiencing chronic pain due to these conditions. The study further has identified the significant common and distinct risk factors attributable to MSDs across 45 to 60 years; 60 years & above and overall Indian adult population (aged ≥ 45 years). This information is highly critical as customised policy interventions can be developed targeting distinctive MSD attributable risk factors for a specific age category of older Indian adults.

### Limitations

The current study faces a few limitations. The diagnosis of MSD was based on self-reported information; therefore chances of reporting errors and recall bias could be there. Further, in the LASI data, only back pain and joint pain were included as MSD and detailed classification on broad spectrum of MSDs were not present. The discrepancies in prevalence at sub-national level in India may be attributed to state specific characteristics and due to some unaccounted factors also. Due to the cross-sectional nature of the study, the risk factors of MSD indicate associational relationships and therefore the causal relationship cannot be sufficiently established. Some important factors such as bone mineral density, which is a critical predictor for MSDs are not included in the LASI survey. Such predictors serve as crucial risk factors and can be included in the next wave of LASI survey for better understanding. Further, certain occupation groups, which have recorded to be significantly associated with MSD presence has not been defined by the LASI survey report. This limits the interventions and policy recommendation for such occupational groups on account of its lack of explicit definition. Furthermore, longitudinal studies based on utilizing the first wave of LASI and its impending second wave are recommended to be conducted to examine the temporal nature of MSD and its predictors.

## Conclusion

This study emphasizes sizable frequency of musculoskeletal disorders (MSDs) among older Indian adults, especially in the age ranges between 45 to 60 and older. The study also discovered that MSDs are more common in women than in men, which may be a result of physiological and anatomical changes brought on by aging and hormonal factors. The incidence of MSDs was significantly influenced by occupation, with professions having higher probabilities of developing MSDs due to the nature of their work involving more stress, physical labour, and trauma. Physical activity and MSDs were discovered to have a complicated association. A higher prevalence of MSDs was linked to more frequent, vigorous physical exercise, while having no physical activity raised the risk as well. Higher BMI was linked to an increased prevalence of MSDs, which is probably caused by unhealthy lifestyle choices such inactivity and sedentary behaviour. The presence of MSDs was also positively associated with hypertension and tobacco use. However, drinking alcohol was linked to a lower occurrence of MSDs, perhaps because of its anti-inflammatory properties.

To address MSDs; preventive measures, early detection, and suitable management techniques are required. The prevalence of MSDs can be decreased, and musculoskeletal health can be improved by addressing modifiable risk factors such physical inactivity, tobacco usage, avoiding workplace risks in occupations requiring manual labour, like- skilled agriculture and fishery workers and elementary workers. In this regard, MSDs prevention management strategies like lifestyle changes (tobacco cessation, dietary management and moderate physical activities); social awareness especially among older adults (> 45 years); incorporating strength training at the workplace can reduce MSDs among physically demanding occupations [37, 38].

Thus, the present study offers a comprehensive analysis of MSDs in India, elucidating their prevalence at sub-national level, exploring the association between MSDs and related risk factors, identifying distinctive attributable risk factors for a specific age category, and providing directions for enhancing patient care. However, future studies are recommended to comprehend the underlying pathophysiological mechanisms and potential confounding variables influencing the occurrence of MSDs.

## Supporting information

S1 Table Prevalence of MSD in the Indian population aged 45–60 years, >60 years and overall. (DOCX)

S2 Table Univariate and multivariable logistic regression of musculoskeletal disorders and various risk factors among the overall population (≥45 years). (DOCX)

S3 Table Univariate and multivariable logistic regression of musculoskeletal disorders and various risk factors among population 45–60 years. (DOCX)

S4 Table Univariate and multivariable logistic regression of musculoskeletal disorders and various risk factors among population >60 years. (DOCX)

S5 Table Significant common and distinctive risk factors of MSD among overall 45–60 years and >60 years population. (DOCX)

S1 Data (CSV)

S2 Data (DTA)
