# Correction: Prevalence and association of musculoskeletal disorders with various risk factors among older Indian adults: Insights from a nationally representative survey

**DOI:** 10.1371/journal.pone.0322356

**Published:** 2025-04-08

**Authors:** Jaya Tiwari, Pritam Halder, Divya Sharma, Uttam Chand Saini, Vineeth Rajagopal, Tanvi Kiran

The first two authors, Jaya Tiwari and Pritam Halder, should be noted as shared first co-authors on this work.

The third and sixth authors, Divya Sharma and Tanvi Kiran, should be noted as shared co-corresponding authors on this work. Divya Sharma’s email address is: divya2809.sharma@gmail.com.

## References

[1] Tiwari J, Halder P, Sharma D, Saini UC, Rajagopal V, Kiran T. Prevalence and association of musculoskeletal disorders with various risk factors among older Indian adults: Insights from a nationally representative survey. PLoS One. 2024;19(10): e0299415. doi: 10.1371/journal.pone.0299415 39441775 PMC11498719

