# Supplementary material for: Prevalence and association of musculoskeletal disorders with various risk factors among older Indian adults: Insights from a nationally representative survey
Source: PLoS One. 2024 Oct 23;19(10):e0299415. doi: 10.1371/journal.pone.0299415 (PMC11498719; doi:10.1371/journal.pone.0299415)
Supplement: S1 Table — (DOCX) [file pone.0299415.s001.docx]

**Supplementary Table 1: Prevalence of MSD in the Indian population aged 45-60 years, >60 years and overall**

| **Variable** | **Prevalence**  **(95% Confidence Interval) (%)** | | | | |
| --- | --- | --- | --- | --- | --- |
|  | **Aged 45-60 years** | **Aged >60 years** | **Male** | **Female** | **Total** |
| **MSD** | 50.8 (50.1-51.5)* | 60.4 (59.3-61.4)* | 48.9 (48.1-49.6)* | 62.3 (61.4-63.3)* | 53.5 (52.9-54.1) |
| **Back pain** | 31.4 (30.8-32.0)* | 35.5 (34.5-36.6)* | 28.6 (27.9-29.3)* | 40.0 (39.1-41.0)* | 32.6 (32.0-33.1) |
| **Joint pain** | 38.7 (38.1-39.4)* | 49.8 (48.7-50.9)* | 37.5 (36.8-38.2)* | 50.1 (49.1-51.0)* | 41.9 (41.3-42.4) |
| **Arthritis** | 5.6 (5.3-6.0)* | 8.7 (8.2-9.4)* | 5.7 (5.3-6.0)* | 8.1 (7.6-8.7)* | 6.5 (6.2-6.8) |
| **Rheumatism** | 3.2 (2.9-3.4)* | 5.2 (4.7-5.7)* | 2.9 (2.8-3.2)* | 5.1 (4.7-5.6)* | 3.7 (3.5-4.0) |
| **Osteoporosis** | 2.1 (1.9-2.3)* | 3.5 (3.1-3.9)* | 2.3 (2.1-2.5)* | 3.0 (2.7-3.4)* | 2.5 (2.3-2.7) |
| **Any bone/**  **joint disease** | 9.9 (9.5-10.3)* | 15.6 (14.9-16.4)* | 9.9 (9.5-10.4)* | 14.5 (13.8-15.2)* | 11.5 (11.1-11.9) |
| -One participant can suffer from one or more than one of the above-mentioned disorders  * p-value<0.05 | | | | | |
