# Supplementary material for: Prevalence and association of musculoskeletal disorders with various risk factors among older Indian adults: Insights from a nationally representative survey
Source: PLoS One. 2024 Oct 23;19(10):e0299415. doi: 10.1371/journal.pone.0299415 (PMC11498719; doi:10.1371/journal.pone.0299415)
Supplement: S2 Table — (DOCX) [file pone.0299415.s002.docx]

**Supplementary Table 2:** **Univariate and multivariable logistic regression of musculoskeletal disorders and various risk factors among the overall population (≥45 years)**

| **Characteristics** | **Participants** | | **Univariate** | | **Multivariable** | |
| --- | --- | --- | --- | --- | --- | --- |
|  | **Total (N=28436)**  **N (%)** | **MSD present**  **(N= 15213)**  **N (%)** | **Crude odds ratio (95% Confidence interval)** | **p-value** | **Adjusted odds ratio (95% Confidence interval)** | **p-value** |
| **Occupation** |  | | | |  | |
| legislators and senior officials | 187 (0.7) | 72 (38.5) | Reference | - | Reference | - |
| professionals | 809 (2.8) | 354 (43.8) | 1.24 (0.90-1.72) | 0.191 | 1.25 (0.90-1.74) | 0.180 |
| technicians and associate professionals | 341 (1.2) | 141 (41.4) | 1.13 (0.78-1.62) | 0.524 | 1.16 (0.80-1.67) | 0.437 |
| clerks | 525 (1.9) | 213 (40.6) | 1.09 (0.77-1.54) | 0.620 | 1.10 (0.78-1.56) | 0.581 |
| service workers and shopkeepers | 2,509 (8.8) | 1,175 (46.8) | 1.41 (1.04-1.91) | 0.028 | 1.45 (1.07-1.97) | 0.018 |
| skilled agriculture and fishery workers | 10,311 (36.3) | 5,632 (54.6) | 1.92 (1.43-2.59) | <0.001 | 2.06 (1.53-2.79) | <0.001 |
| Craft and related trade worker | 827 (2.9) | 383 (46.3) | 1.38 (1.00-1.91) | 0.053 | 1.44 (1.04-2.00) | 0.028 |
| plant and machine operator | 571 (2.0) | 233 (40.8) | 1.10 (0.78-1.54) | 0.577 | 1.14 (0.81-1.61) | 0.441 |
| elementary occupations | 5,468 (19.2) | 3,160 (57.8) | 2.19 (1.62-2.95) | <0.001 | 2.30 (1.70-3.11) | <0.001 |
| Others | 6,888 (24.2) | 3,850 (55.9) | 2.02 (1.50-2.73) | <0.001 | 2.14 (1.59-2.90) | <0.001 |
| **Employment Duration (years) documented** |  | | | |  | |
| <5 | 1486 (5.2) | 778 (52.4) | Reference | - | Reference | - |
| >5 | 26950 (94.8) | 14435 (53.6) | 1.10 (0.95-1.17) | 0.064 | 1.03 (0.93-1.15) | 0.533 |
| **Vigorous Physical activity** |  | | | |  | |
| Everyday | 10,945 (38.5) | 5912 (54.0) | Reference | - | Reference | - |
| More than once / week | 3,044 (10.7) | 1730 (56.8) | 1.12 (1.03-1.21) | 0.006 | 1.12 (1.03-1.21) | 0.007 |
| Once / week | 1,333 (4.7) | 708 (53.1) | 0.96 (0.86-1.08) | 0.533 | 0.98 (0.87-1.10) | 0.693 |
| 1-3 times /month | 1,849 (6.5) | 1029 (55.6) | 1.07 (0.97-1.18) | 0.192 | 1.07 (0.97-1.18) | 0.190 |
| Never | 11265 (39.6) | 5834 (51.8) | 0.91 (0.87-0.96) | 0.001 | 0.93 (0.88-0.98) | 0.010 |
| **BMI** |  | | | |  | |
| <18.5 | 5,329 (18.7) | 2,810 (52.7) | Reference | - | Reference | - |
| 18.5-22.9 | 11601 (40.8) | 6,241 (53.8) | 1.04 (0.98-1.11) | 0.196 | 1.06 (0.99-1.13) | 0.106 |
| 23-24.9 | 4378 (15.4) | 2,260 (51.6) | 0.96 (0.88-1.04) | 0.277 | 0.99 (0.91-1.07) | 0.738 |
| 25-29.9 | 5730 (20.2) | 3,135 (54.7) | 1.08 (1.01-1.17) | 0.037 | 1.13 (1.05-1.23) | 0.002 |
| >30 | 1398 (4.9) | 767 (54.9) | 1.09 (0.97-1.23) | 0.155 | 1.13 (0.99-1.28) | 0.056 |
| **Currently Diabetic** |  | | | |  | |
| No | 25781 (90.7) | 13785 (53.5) | Reference | - | Reference | - |
| Yes | 2655 (9.3) | 1428 (53.8) | 1.01 (093-1.10) | 0.756 | - | - |
| **Currently Hypertensive** |  | | | |  | |
| No | 22459 (78.9) | 11561 (51.5) | Reference | - | Reference | - |
| Yes | 5977 (21.1) | 3652 (61.1) | 1.48 (1.39-1.57) | <0.001 | 1.53 (1.44-1.63) | <0.001 |
| **Tobacco usage** |  | | | |  | |
| No | 15225 (53.5) | 8080 (53.1) | Reference | - | Reference | - |
| Yes | 13211 (46.5) | 7133 (53.9) | 1.04 (0.99-1.09) | 0.120 | 1.04 (0.99-1.09) | 0.159 |
| **Alcohol consumption** |  | | | |  | |
| No | 20895 (73.5) | 11199 (53.6) | Reference | - | Reference | - |
| Yes | 7541 (26.5) | 4014 (53.5) | 0.99 (1.12-1.19) | 0.583 | - | - |
| Goodness of fit statistics  The analysis predicted probabilities for those with the presence of MSD.  The Omnibus Tests of Model Coefficients gives a Chi-Square of 529.62 (p<0.001).  The pseudo R^2^ value = 0.0135; Predictive model classification accuracy = 56.02% | | | | | | |
