# Supplementary material for: Prevalence and association of musculoskeletal disorders with various risk factors among older Indian adults: Insights from a nationally representative survey
Source: PLoS One. 2024 Oct 23;19(10):e0299415. doi: 10.1371/journal.pone.0299415 (PMC11498719; doi:10.1371/journal.pone.0299415)
Supplement: S3 Table — (DOCX) [file pone.0299415.s003.docx]

**Supplementary Table 3:** **Univariate and multivariable logistic regression of musculoskeletal disorders and various risk factors among population 45-60 years**

| **Characteristics** | **Participants** | | **Univariate** | | **Multivariable** | |
| --- | --- | --- | --- | --- | --- | --- |
|  | **Age 45-60 years (N=20396)**  **N (%)** | **MSD present**  **(N= 10360)**  **N (%)** | **Crude odds ratio (95% Confidence interval)** | **p-value** | **Adjusted odds ratio (95% Confidence interval)** | **p-value** |
| **Occupation** |  | | | |  | |
| legislators and senior officials | 150 (0.7) | 58 (38.7) | Reference | - | Reference | - |
| professionals | 691 (3.4) | 290 (42.0) | 1.15 (0.80-1.65) | 0.457 | 1.15 (0.80-1.66) | 0.445 |
| technicians and associate professionals | 298 (1.5) | 122 (40.9) | 1.10 (0.74-1.64) | 0.643 | 1.14 (0.76-1.70) | 0.537 |
| clerks | 449 (2.2) | 174 (38.8) | 1.00 (0.69-1.47) | 0.985 | 1.01 (0.69-1.48) | 0.957 |
| service workers and shopkeepers | 1,838 (9.0) | 824 (44.8) | 1.29 (0.92-1.81) | 0.145 | 1.33 (0.94-1.88) | 0.104 |
| skilled agriculture and fishery workers | 6,830 (33.5) | 3560 (52.1) | 1.73 (1.24-2.41) | 0.001 | 1.84 (1.31-2.57) | <0.001 |
| Craft and related trade worker | 612 (3.0) | 273 (44.6) | 1.28 (0.89-1.84) | 0.189 | 1.35 (0.93-1.95) | 0.111 |
| plant and machine operator | 482 (2.4) | 197 (40.9) | 1.10 (0.75-1.60) | 0.631 | 1.14 (0.78-1.66) | 0.496 |
| elementary occupations | 3,896 (19.1) | 2119 (54.4) | 1.89 (1.35-2.64) | <0.001 | 1.99 (1.42-2.78) | <0.001 |
| Others | 5,150 (25.3) | 2743 (53.3) | 1.81 (1.30-2.52) | <0.001 | 1.91 (1.36-2.67) | <0.001 |
| **Employment Duration (years) documented** |  | | | |  | |
| <5 | 1038 (5.1) | 511 (49.2) | Reference | - | Reference | - |
| >5 | 19358 (94.9) | 9849 (50.9) | 1.07 (0.94-1.21) | 0.301 | - | - |
| **Vigorous Physical activity** |  | | | |  | |
| Everyday | 8140 (39.9) | 4209 (51.7) | Reference | - | Reference | - |
| More than once / week | 2208 (10.8) | 1,199 (54.3) | 1.11 (1.01-1.21) | 0.030 | 1.11 (1.01-1.22) | 0.032 |
| Once / week | 964 (4.7) | 486 (50.4) | 0.95 (0.83-1.08) | 0.448 | 0.96 (0.84-1.10) | 0.594 |
| 1-3 times /month | 1290 (6.3) | 689 (53.4) | 1.07 (0.95-1.21) | 0.255 | 1.07 (095-1.21) | 0.266 |
| Never | 7794 (38.2) | 3,777 (48.5) | 0.88 (0.83-0.93) | <0.001 | 0.89 (0.84-0.95) | 0.001 |
| **BMI** |  | | | |  | |
| <18.5 | 3268 (16.0) | 1620 (49.6) | Reference | - | Reference |  |
| 18.5-22.9 | 8163 (40.0) | 4158 (50.9) | 1.06 (0.97-1.15) | 0.187 | 1.07 (0.99-1.16) | 0.102 |
| 23-24.9 | 3345 (16.4) | 1652 (49.4) | 0.99 (0.90-1.09) | 0.881 | 1.02 (0.93-1.13) | 0.675 |
| 25-29.9 | 4464 (21.9) | 2326 (52.1) | 1.11 (1.01-1.21) | 0.028 | 1.16 (1.05-1.27) | 0.003 |
| >30 | 1156 (5.7) | 604 (52.3) | 1.11 (0.97-1.27) | 0.118 | 1.14 (0.99-1.31) | 0.068 |
| **Currently Diabetic** |  | | | |  | |
| No | 18636 (91.4) | 9450 (50.7) | Reference | - | Reference | - |
| Yes | 1760 (8.6) | 910 (51.7) | 1.04 (0.94-1.15) | 0.424 | - | - |
| **Currently Hypertensive** |  | | | |  |  |
| No | 16491 (80.9) | 8055 (48.8) | Reference | - | Reference | - |
| Yes | 3905 (19.2) | 2305 (59.0) | 1.51 (1.41-1.62) | <0.001 | 1.56 (1.45-1.68) | <0.001 |
| **Tobacco usage** |  | | | |  | |
| No | 11306 (55.4) | 5695 (50.4) | Reference | - | Reference | - |
| Yes | 9090 (44.6) | 4665 (51.3) | 1.04 (0.98-1.10) | 0.178 | 1.06 (1.01-1.12) | 0.046 |
| **Alcohol consumption** |  | | | |  | |
| No | 15022 (73.7) | 7686 (51.2) | Reference | - | Reference | - |
| Yes | 5374 (26.3) | 2674 (49.7) | 1.05 (1.02-1.08) | 0.004 | 0.92 (0.86-0.98) | 0.010 |
| Goodness of fit statistics  The analysis predicted probabilities for those with the presence of MSD  The Omnibus Tests of Model Coefficients gives a Chi-Square of 366.78 (p<0.001).  The pseudo R^2^ value = 0.0130; Predictive model classification accuracy = 54.80% | | | | | | |
