# Supplementary material for: Prevalence and association of musculoskeletal disorders with various risk factors among older Indian adults: Insights from a nationally representative survey
Source: PLoS One. 2024 Oct 23;19(10):e0299415. doi: 10.1371/journal.pone.0299415 (PMC11498719; doi:10.1371/journal.pone.0299415)
Supplement: S4 Table — (DOCX) [file pone.0299415.s004.docx]

**Supplementary Table 4:** **Univariate and multivariable logistic regression of musculoskeletal disorders and various risk factors among population >60 years**

| **Characteristics** | **Participants** | | **Univariate** | | **Multivariable** | |
| --- | --- | --- | --- | --- | --- | --- |
|  | **Age >60 years (N=8040)**  **N (%)** | **MSD present (N= 4853)**  **N (%)** | **Crude odds ratio (95% Confidence interval)** | **p-value** | **Adjusted odds ratio (95% Confidence interval)** | **p-value** |
| **Occupation** |  | | | |  | |
| legislators and senior officials | 37 (0.5) | 14 (37.8) | Reference | - | Reference | - |
| professionals | 118 (1.5) | 64 (54.2) | 1.95 (0.91-4.15) | 0.084 | 2.08 (0.97-4.45) | 0.060 |
| technicians and associate professionals | 43 (0.5) | 19 (44.2) | 1.30 (0.53-3.19) | 0.566 | 1.39 (0.56-3.42) | 0.479 |
| clerks | 76 (1.0) | 39 (51.3) | 1.73 (0.78-3.86) | 0.180 | 1.85 (0.82-4.14) | 0.136 |
| service workers and shopkeeper | 671 (8.4) | 351 (52.3) | 1.80 (0.91-3.56) | 0.090 | 1.96 (0.99-3.90) | 0.054 |
| skilled agriculture and fishery workers | 3,481 (43.3) | 2072 (59.5) | 2.42 (1.24-4.71) | 0.010 | 2.85 (1.45-5.60) | 0.002 |
| Craft and related trade worker | 215 (2.7) | 110 (51.2) | 1.72 (0.84-3.52) | 0.137 | 1.90 (0.92-3.91) | 0.081 |
| plant and machine operator | 89 (1.1) | 36 (40.5) | 1.12 (0.51-2.45) | 0.785 | 1.22 (0.55-2.71) | 0.618 |
| elementary occupations | 1,572 (19.6) | 1041 (66.2) | 3.22 (1.64-6.31) | 0.001 | 3.66 (1.86-7.22) | <0.001 |
| Others | 1,738 (21.6) | 1107 (63.7) | 2.88 (1.47-5.64) | 0.002 | 3.32 (1.69-6.54) | 0.001 |
| **Employment Duration (years) documented** |  | | | |  | |
| <5 | 448 (5.6) | 267 (59.6) | Reference | - | Reference | - |
| >5 | 7592 (94.4) | 4586 (60.4) | 1.03 (0.85-1.26) | 0.734 | - | - |
| **Vigorous Physical activity** |  | | | |  | |
| Everyday | 2805 (34.9) | 1,703 (60.7) | Reference | - | Reference | - |
| More than once / week | 836 (10.4) | 531 (63.5) | 1.13 (0.96-1.32) | 0.144 | 1.12 (0.96-1.32) | 0.159 |
| Once / week | 369 (4.6) | 222 (60.2) | 0.98 (0.78-1.22) | 0.839 | 0.97 (0.77-1.21) | 0.767 |
| 1-3 times /month | 559 (7.0) | 340 (60.8) | 1.01 (0.83-1.21) | 0.961 | 0.99 (0.82-1.20) | 0.941 |
| Never | 3471 (43.2) | 2,057 (59.3) | 0.94 (0.85-1.04) | 0.244 | 0.93 (0.84-1.03) | 0.169 |
| **BMI** |  | | | |  | |
| <18.5 | 2061 (25.6) | 1190 (57.7) | Reference | - | Reference |  |
| 18.5-22.9 | 3438 (42.8) | 2083 (60.6) | 1.13 (1.001-1.26) | 0.037 | 1.12 (1.01-1.25) | 0.047 |
| 23-24.9 | 1033 (12.9) | 608 (58.9) | 1.05 (0.90-1.22) | 0.552 | 1.07 (0.91-1.25) | 0.417 |
| 25-29.9 | 1266 (15.8) | 809 (63.9) | 1.30 (1.12-1.50) | <0.001 | 1.34 (1.16-1.56) | <0.001 |
| >30 | 242 (3.0) | 163 (67.4) | 1.51 (1.14-2.00) | 0.004 | 1.60 (1.19-2.24) | 0.002 |
| **Currently Diabetic** |  | | | |  | |
| No | 7145 (88.9) | 4335 (60.7) | Reference | - | Reference | - |
| Yes | 895 (11.1) | 518 (57.9) | 0.89 (0.77-1.03) | 0.207 | - | - |
| **Currently Hypertensive** |  | | | |  |  |
| No | 5968 (74.2) | 3506 (58.8) | Reference | - | Reference | - |
| Yes | 2072 (25.8) | 1347 (65.0) | 1.30 (1.18-1.45) | <0.001 | 1.31 (1.17-1.45) | <0.001 |
| **Tobacco usage** |  | | | |  | |
| No | 3919 (48.7) | 2385 (60.9) | Reference | - | Reference | - |
| Yes | 4121 (51.3) | 2468 (59.9) | 0.96 (0.88-1.05) | 0.375 | - | - |
| **Alcohol consumption** |  | | | |  | |
| No | 5873 (73.1) | 3513 (59.8) | Reference | - | Reference | - |
| Yes | 2167 (26.9) | 1340 (61.8) | 1.09 (0.98-1.20) | 0.100 | 1.08 (0.98-1.20) | 0.127 |
| Goodness of fit statistics  The analysis predicted probabilities for those with the presence of MSD  The Omnibus Tests of Model Coefficients gives a Chi-Square of 363.23 (p<0.001).  The pseudo R2 value = 0.0128  Predictive model classification accuracy = 54.88% . | | | | | | |
