# Supplementary material for: Prevalence and association of musculoskeletal disorders with various risk factors among older Indian adults: Insights from a nationally representative survey
Source: PLoS One. 2024 Oct 23;19(10):e0299415. doi: 10.1371/journal.pone.0299415 (PMC11498719; doi:10.1371/journal.pone.0299415)
Supplement: S5 Table — (DOCX) [file pone.0299415.s005.docx]

**Supplementary Table 5: Significant common and distinctive risk factors of MSD among overall 45-60 years and >60 years population**

| **Common risk factor** | **Overall Population**  **(aOR, 95% CI)** | **Population aged**  **45-60 years**  **(aOR, 95% CI)** | **Population aged**  **>60 years**  **(aOR, 95% CI)** |
| --- | --- | --- | --- |
| **Occupation** |  |  |  |
| professionals | - | - | - |
| technicians and associate professionals | - | - | - |
| clerks | - | - | - |
| service workers and shopkeepers | 1.45 (1.07-1.97) | - | - |
| skilled agriculture and fishery workers | 2.06 (1.53-2.79) | 1.84 (1.31-2.57) | 2.85 (1.45-5.60) |
| Craft and related trade worker | 1.44 (1.04-2.00) | - | - |
| plant and machine operator | - | - | - |
| elementary occupations | 2.30 (1.70-3.11) | 1.99 (1.42-2.78) | 3.66 (1.86-7.22) |
| Others | 2.14 (1.59-2.90) | 1.91 (1.36-2.67) | 3.32 (1.69-6.54) |
| **Employment Duration (>5years) documented** | - | - | - |
| **Vigorous Physical activity** |  |  |  |
| More than once / week | 1.12 (1.03-1.21) | 1.11 (1.01-1.22) | - |
| Once / week | - | - | - |
| 1-3 times /month | - | - | - |
| Never | 0.93 (0.88-0.98) | 0.89 (0.84-0.95) | - |
| **BMI** |  |  |  |
| 18.5-22.9 | - | - | 1.12 (1.01-1.25) |
| 23-24.9 | - | - | - |
| 25-29.9 | 1.13 (1.05-1.23) | 1.16 (1.05-1.27) | 1.34 (1.16-1.56) |
| >30 | - | - | 1.60 (1.19-2.24) |
| **Currently Diabetic** | - | - | - |
| **Currently Hypertensive** | 1.53 (1.44-1.63) | 1.56 (1.45-1.68) | 1.31 (1.17-1.45) |
| **Tobacco usage** | - | 1.06 (1.01-1.12) | - |
| **Alcohol consumption** | - | 0.92 (0.86-0.98) | - |
